# Coherent Investigation on a Smart Kinetic Wooden Façade Based on Material Passport Concepts and Environmental Profile Inquiry

**DOI:** 10.3390/ma14143771

**Published:** 2021-07-06

**Authors:** Amjad Almusaed, Ibrahim Yitmen, Asaad Almsaad, İlknur Akiner, Muhammed Ernur Akiner

**Affiliations:** 1 Department of Construction Engineering and Lighting Science, Jönköping University, 551 11 Jönköping, Sweden; ibrahim.yitmen@ju.se; 2 Department of Engineering and Chemical Sciences, Karlstad University, 651 88 Karlstad, Sweden; asaad.almssad@kau.se; 3 Department of Architecture, Akdeniz University, Antalya 07058, Turkey; ilknurakiner@akdeniz.edu.tr; 4 Vocational School of Technical Sciences, Akdeniz University, Antalya 07058, Turkey; ernurakiner@akdeniz.edu.tr

**Keywords:** double skin, environmental profile, material passport, kinetic façade, wooden façade

## Abstract

Wood is one of the most fully renewable building materials, so wood instead of non-renewable materials produced from organic energy sources significantly reduces the environmental impact. Construction products can be replenished at the end of their working life and their elements and components deconstructed in a closed-loop manner to act as a material for potential construction. Materials passports (MPs) are instruments for incorporating circular economy principles (CEP) into structures. Material passports (MPs) consider all the building’s life cycle (BLC) steps to ensure that it can be reused and transformed several times. The number of reuse times and the operating life of the commodity greatly influence the environmental effects incorporated. For a new generation of buildings, the developing of an elegant kinetic wooden façade has become a necessity. It represents a multidisciplinary region with different climatic, fiscal, constructional materials, equipment, and programs, and ecology-influencing design processes and decisions. Based on an overview of the material’s environmental profile (MEP) and material passport (MP) definition in the design phase, this article attempts to establish and formulate an analytical analysis of the wood selection process used to produce a kinetic façade. The paper will analyze the importance of environmentally sustainable construction and a harmonious architectural environment to reduce harmful human intervention on the environment. It will examine the use of wooden panels on buildings’ façades as one solution to building impact on the environment. It will show the features of the formation of the wooden exterior of the building. It will also examine modern architecture that enters into a dialogue with the environment, giving unique flexibility to adapt a building. The study finds that new buildings can be easily created today. The concept of building materials passport and the environmental selection of the kinetic wooden façade can be incorporated into the building design process. This will improve the economic and environmental impact of the building on human life.

## 1. Introduction

The construction industry accounts for more than 30 percent of the extraction of natural resources and 25 percent of the world’s solid waste since the construction industry mostly follows a sequential business paradigm of “taking, manufacturing, disposing of,” using and disposing of goods at the end of their lives, as they are put together for the one-off and not reused [1]. The industry has been changing its paradigms over recent decades by implementing a circular economy model to maintain closed-loop materials with the highest capacity for minimizing waste production and resource mining for the construction industry [2]. In particular, it will continue to be a paradigm change in the industry as a whole. According to the Ellen MacArthur Foundation (EMF), which works in accelerating the transition to a circular economy, the end-of-life goods and building materials, and components, can be reused, used as resource banks for new buildings, and retained as the circular economy’s general perspective in a closed-loop segment or material [3]. However, for greater market acceptance, this definition requires knowledge development and tools [4], particularly in the building sector, in which innovational engineering design takes more time [5] since buildings are always one-of-a-kind designs together with the wide supply chain, which adds to the difficulty [6]. The design of a dynamic (kinetic) façade is one of the most interesting solutions in the structure, adapting to changes in the outside environment.

The kinetic façade is a breakthrough in buildings’ architecture [7]. For the Scandinavian region, kinetic façades are a new trend, in which they can currently be counted on the fingers of a hand [8]. If we talk about greening, today it is relevant all over the world; there are more and more green areas in large cities. Dynamic façades adapt the space according to the needs of the people.

Buildings with variable façades can certainly be called a new era in architecture. The kinetic façade is a breakthrough trend in building architecture. The façade is in motion; here is a more accurate description of it: A kinetic façade is a building cladding in constant motion from an engineering perspective under the influence of nature or mechanics forces [9,10]; this is a constantly changing façade pattern [11]. Therefore, selecting appropriate materials is a critical and meticulous step to achieve the building façade design’s desired effect [12].

Different materials can convey different feelings on the exterior of the building. A tight grasp of the materials’ properties can create an extraordinary and intriguing façade effect when using materials. The influence of materials and craftsmanship on the design of building façades is very important. In recent year primitive natural building materials to the widely used building materials such as woods, stones, and reeds have formed the architectural style characteristics of various periods.

Although kinetic façades are quite new, the types they take are already very diverse. Every style, including triangular elements to giant sunshades, wooden frames, and projected animations, serves a specific function and takes on a distinct form [13].

Consisting of qualitative knowledge, also the quantitative database of a structure’s material property, the material passport (MP) presents components found in designs and demonstrates their recyclability and environmental effects [14]. Today, the structure of kinetic wooden materials for the façade in a new generation of building conception is supported by strong arguments, innovations, and improvements introduced in the recent period, helping promote ecological material for new construction worldwide [15]. The research in the kinetic wooden façade as part of sustainable environment buildings arouses great interest in the construction sector. Thus, we will see opportunities to develop wood construction research considerably at our universities in the next few years. It allows us to establish construction technology as a research subject at our institutions and strengthen the relation with city municipalities in usable form. BEAT 2000 is a systematic methodology of environmental assessment and measurement of environmental impacts of building materials in their life cycles [14] based on the SBI (DK) simulation programmer used to calculate the environmental profile.

The objective of this research is to address questions such as:

- What is the role of the kinetic façade in modern architecture today?
- How does the kinetic wooden façade contribute to modern building conception?
- Why is the material passport system required in creating the ecological kinetic façade?
- What is the evaluation of the environmental profile impact on the wooden façade?

The analysis will review the most appropriate building material passports used in a smart wooden kinetic façade to confirm the environment’s natural material. First, this research attempts to classify the criteria that have an especially strong effect on material selection through material passports. The second goal is to assess various architecture systems and their basic requirement in light of the chosen solution requirements. Finally, this investigation will focus on using wooden and ecological materials to build façade models and technology.

The research will provide an efficient material passport (MP) and a smart technology for innovative kinetic wooden façade architecture, where modern technical components can be integrated into façade elements. Almost all façade materials and systems are suitable for use combined with different kinds of wood to express the designer’s creation intent and match the environment [16]. Temperature and humidity can be effectively adjusted under proper insulation by cooperating with board and membrane materials with different building physical properties [16]. The building’s energy consumption can be reduced to the lowest level under the premise of ensuring the indoor environment’s comfort. Ruggiero et al. [17] supposed that over the last 40 years, reforming in the building sector has had an extraordinary evolution, which has led, from the intuition of the possibility to import models and methodologies from the industrial sector an environmental and safety building component. The most usable material in history was wood. Waste-free properties, thermal efficiency, durability, original texture, an advantage in handling, and several additional wood attributes contribute to a comfortable and relaxing living environment [18]. Munir et al. [19] conclude that the wood’s raw, brittle, and moisture-absorbing properties are often misunderstood because of its organic, porous, and moisture-absorbing surface, where the organic nature of wood makes it environment-friendly. Regarding the environmental and energy considerations, the utilization of wood in design is regarded as the best material for addressing these problems. It is no coincidence that existing structures were made of solid wood to enhance their structural and architectural qualities. Wood has lower thermal conductivity than many construction materials and is ideal for an energy-efficient design [20]. Wood buildings behave similarly to passive solar houses by absorbing and storing heat in the wood pulp [20]. Sekularac et al. [21] think that the wood is an element of façade cladding in modern architecture, and the research is intended to expand knowledge of the possibilities and limitations and create the foundation for their correct wider use. Sekularac et al. [21] consider that the wood and wood-based components of the building are used as double skin layers in the façade, where the temperature, solar radiation, and wind have a certain impact on the architectural presence of a structure. The wooden façade has been common material used in conjunction with ecological building materials to express the wooden structure’s natural texture directly. Solovev [22] believes that the construction material’s choosing process is the most important decision and has long-term consequences for the structure’s owner.

Zhukov et al. [23] are confident that the concept of an objective selection of construction materials should include requirements for materials, building systems in which these materials are used, work technology, architectural and planning solutions, and engineering support systems. Khoshnava et al. [24] consider that the important factor in selecting building material is to be an environmentally safe material for a toxic-free environment. That will have a positive impact on humans and the environment. Lawson [25] confirms that the safety assessment considers the impact of material on the environment in all its life cycles. Haupt and Hellweg [26] think the most important indicators of the material’s environmental friendliness are the possibility of recycling, energy consumption, environmental friendliness of production, and operational characteristics. Wood occupies a special and important place among building materials, having an undoubted priority in “sustainable architecture” [27].

Assefa et al. [28] think that after experiencing wind and rain and other climatic conditions, the façade presents a special texture without losing its function. The effect of a building on the atmosphere is determined by the materials used and the energy sources used. Wooden kinetic façades may be used with or without surface protection. Shahda [29] suggests that the change in building technology was from traditional building technology to smart, sustainable architecture, expanded use of environmentally safe materials in design, and environmentally friendly wood conservation solutions in his paper. Fakourian and Asefi [30] consider that buildings with a kinetic wooden façade are climate-smart, not least because they bind carbon dioxide and prevent it from being released.

At the “World Congress of Architects” in 1993, Thayer [31] proposed that the architectural climate in general and structures are among the most significant components in the detrimental human impact on the natural environment in the “Declaration Interdependence for a Sustainable Future”.

## 2. Façade Analysis within the Thematic Area

### 2.1. Wooden Façade and Building Material Passport (BMP)

Choosing passport materials for creating a competent architectural element with suitability for a building component or category becomes required. It depends on creating an objective. There is still no clear technical regulation within the EU that would comprehensively regulate the building material passport (BMP), which describes the suitability, quality, and safety requirements in different buildings and programs. All building materials require a mandatory assessment of compliance with competency, suitability, and safety requirements. A structured description of building materials allows more successful working in the ABC industry, developing the concept of efficiency and bio-economy in the construction sector [32]. The wide range of building materials put into circulation at the moment is so extensive that it is often impossible to do without difficulties in determining the composition of the mandatory accompanying classifications and information. Quality and material classification are primary details characterizing and describing building materials’ products [33]. To some extent, a technical passport of products, which is an integral part of the accompanying information, can act as a passport for the quality of building materials. Still, this phrase is generally understood as any material description that testifies building materials’ quality, suitability, and safety.

Companies’ economic models are transformed as circular economy practices that are implemented. If data are systematized and optimized, it becomes easier to adapt, add value, and implement energy-efficient and recycling initiatives in the building industry. BMPs are instruments for incorporating the circular economy into residential design. Creating more effective and resilient ecosystems, they would be crucial in preserving and delivering knowledge to consumers in company supply chains. Resources’ worth and useful life are maintained, repaired, or even improved by locating them in a database, transferring them, and reusing them. Munaro et al. [34] propose a BMP model for Brazil’s wood-frame structure, including general awareness, security, preservation, use, service, installation directions, reuse, and product support history. Centered on Munaro’s BMP concept, this analysis proposes a MP for wood façades.

### 2.2. Wooden Façade and Environmental Profile Analysis (EPA)

Environmental considerations are on the way to becoming an integral part of the design process when creating the world’s architecture and construction. In the 1990s of the last century, the environment finally came on architecture’s main agenda [35]. However, the problem is not infrequently handled at a somewhat naive level, focusing on the signal value rather than concrete environmental results. The environment is something with nature; so-called natural materials are preferred when environmentally friendly. In reality, all materials, even plastic, come from nature. Simultaneously, all common building materials have undergone a processing process, i.e., they are not natural in the sense of the original [14]. However, it is often meant as wood or green areas. Philosophically, it is about different ways of understanding the world.

On the other hand, the romantic wants to emphasize the sensual and the thoughts and feelings it triggers in the viewer. The engineer and the architect fill different roles in the construction. The engineer is educated in a scientific tradition and must strive for “objective truth”. It is something of a mouthful about contemporary construction’s multifaceted reality, even when limited to the physical field. Therefore, the engineer must specialize. With the specialization, the overview and understanding of the built whole are weakened, just as the risk of using agreed-upon terminology increases. It requires comparing the material-related environmental impacts with the selected building material passport. It is necessary to enhance the choice process of material and its overall environmental impact to get objective results to use numbers, diagrams, words, and pictures to describe the topic.

## 3. Kinetic Façade Role in Modern Building Design

An ecological kinetic façade with a new modular building structure means a façade system with a modular preassembled construction adjusted to various required conditions in different sites and positions [36].

Kinetic architecture is the art and science of constructing buildings so that structural elements can move relative to each other without disrupting the building’s overall integrity. Kinetic factors affect how the building panels move, fold, rotate, and transform, solving various climatic and aesthetic problems [37]. The visual transformation in this architecture direction is not hidden between the internal engineering communications [38]. The process of changing the façade of kinetic buildings is visible to everyone—if you need to hide the room from the sun, then the whole house will “take” this in. In the early 20th century, architects began to explore the possibility of introducing kinetics elements into buildings. The understanding was formed that movement in architecture can be produced with engines mechanically or using people, air, water, and other kinetic forces. For example, the wooden kinetic façade can include massive wooden elements supported by separate frames from the outer wall. According to the façade orientations, the façade’s kinetic aspects are programmed to reduce sunlight’s influence.

Every year, dozens of new original designs of dynamic façades and building envelopes appear globally, allowing in time to change buildings’ appearance and perform several additional functions to regulate lighting, heat protection of a building, and air exchange of premises [39]. Architectural structures are considered static objects, but most have special equipment that lets the building adjust to varying settings. Controls and digital technology are revolutionizing our lives, automating nearly every aspect of our lives. These innovations are increasingly being used in building architecture and construction [40]. These involve movable partitions, walls, active ventilation openings, curtains, screens, and blinds, and the mechanized sections of the system that enable it to respond to changing external environmental conditions and human behavior. Kinetic façades as controlled dynamic structures are also found in modern building systems in most countries [41]. The change in the position of these structures is due to certain factors: if it is necessary to increase energy efficiency, when the temperature inside the building fluctuates (i.e., based on the microclimate of the room), when climatic conditions change, for artistic reasons, which attracts more people to structures and spaces. The era of responsive building components and dynamic architecture that respond to consumer demands rapidly evolved from the early 20th century to the last transformable façade erected based on algorithmic control that relies on climate data and sunlight. These responsive components are high-tech systems that use networked sensors and actuators to monitor environmental parameters and automate functional building elements’ control.

### 3.1. How Does the Smart Kinetic Wooden Façade (SKWF) Contribute to Modern Building Conception?

A smart, efficient kinetic wooden façade is a high-tech project, where a high-tech product, in general, is a data processing object, with several interactive functions. The work will contribute to reaching the global goals for sustainable development. Relevant objectives are minimal resource usage throughout the whole product life cycle, increased prosperity by making technology and products available worldwide, and life-long learning in the industry through flexible education alternatives [21]. The importance of a smart kinetic wooden façade (SKWF) as a system that comes directly in energy performances and healthy buildings is high, where it becomes required to develop this element according to EU standards. The study will investigate the essential effect of using this system in the city façade and the environmental benefit. The study will also open a way to activate the passport concept in the design process as a sustainability tool in building design conception. Wooden kinetic façades can be largely prefabricated.

Modern architecture and technologies guarantee a high degree of precision, a basic requirement for quality construction. The assembly is thus considerably reduced, and the construction period is much shorter. Wood is a natural material. Untreated, it withstands changes in color and surface structure due to climatic influences. The natural color of fresh wood is not durable to any wood species used outdoors [42]. These changes, however, do not affect the strength of the wood in any way but are rather signs of aging of the living material. As a material for constructing façades, wood will be suitable for all types of facilities, including residential buildings. By choosing the type of wood, which corresponds to the environmental profile, and the installation and the surface treatment, the wooden façade can be completely customized, even in colors. Thus, no two wooden façades are alike. The materials selected for the wooden façade can be a special environmental characteristic. The benefits of using environmentally friendly construction materials include practical recycling choices and wood as the building material with the lowest carbon footprint [43]. The new type of ecological materials and technologies appear that make it possible to embody the most daring ideas because non-standard solutions increase the urban environment’s aesthetic appeal. All types of wood used for façades have a long life, and in the end, they are easy to recycle, as described in the environmental profile factors.

### 3.2. Bionic and Bioclimatic Concepts in the Adaptive Ecological Kinetic Façade

Dynamic façades adapt the building to the time of day, weather, and light level. The building lives and exists as part of nature, wakes up at dawn, protects residents on a hot afternoon from bright sunlight, saves energy, and even replenishes its reserves. New systems are currently being actively developed to cover a building from excessive sun and regulate its temperature. Bionics is a growing industry in architecture and construction, and many bio-inspired adaptable façades have gone from concept to reality [44]. It is necessary to establish a more comprehensive, systematic, and rational “transfer” process from nature to the enclosing structures to achieve a thorough application of bionics in architecture, potentially influencing the efficiency of life. Adapting the building envelope to the external climate and user requirement and providing the desired indoor temperature can be learned from nature. Bio-adaptive enclosing structures have great potential in reducing energy consumption and providing a comfortable operating environment [45]. Biological adaptation is the ability of a system to adapt, that is, to meet specified requirements, including when environmental conditions change. Building shells are enclosing structures that can independently react to changes in their atmosphere, such as solar radiation, wind speed and direction, air temperature, and precipitation [46]. As a result, when compared to conventional static buildings, energy demand can be reduced because useful energy sources are only used when they are needed.

Bio-adaptable façades act as a kind of climate mediator between comfort requirements and environmental conditions. Façades with the built-in function of bio-adaptiveness can be designed directly for a specific user. The investigation on the systems of bio-adaptive kinetic façades can be based on various world experiences, where it can highlight the latest trends in using this type of façade in modern buildings and projects. The hypothesized adaptive kinetic façade concept is that this system’s application and viability are possible in the climate of north Europa. The outer shell will open and close depending on weather conditions, regulate the temperature and humidity, and create the necessary ventilation. Thus, an ideal microclimate will be created and maintained at any time of the year, regardless of climatic conditions.

### 3.3. Macro-Climatic Action and Wooden Façade Reaction

**Figure 1:**
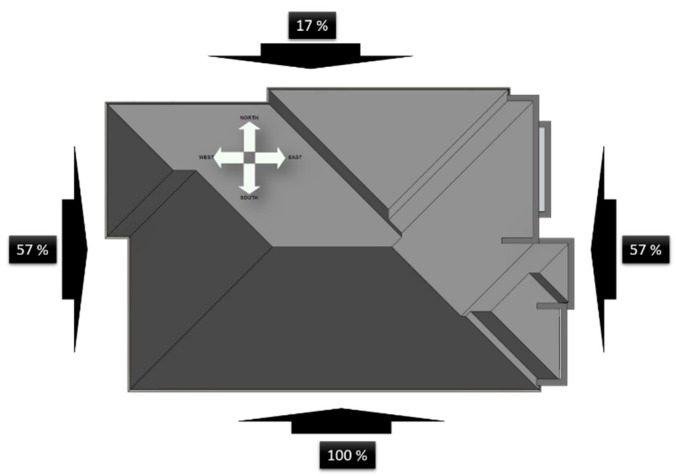
The relative proportion of the energy gained from the outer climate on the building walls from different sides of the world [48].

An increase in wood moisture content above 20–23% inevitably increases fungal attack risk [43]. With drops in humidity and temperature (when the weather changes), wood deforms. Its shrinkage and swelling, alternating, lead to warping and cracking through which water enters the wood structure [47]. Ultraviolet radiation is a destroyer of wood lignin, which binds cellulose and is the main building substance. The primary signs are wood darkening. With longer exposure to the sun, the wood acquires a gray color; small cracks appear in it, and water accumulates (precipitation), which gives an impetus for the reproduction of fungi or mold. Materials for protecting the façade surfaces of wooden houses from atmospheric influences must be elastic and resistant to external forces. On another hand, the building orientation plays an important role in the relative proportion of the energy gained from the outer climate as shown in Figure 1.

## 4. Wooden Materials Use in Wooden Façade

The European timber industry is strongly committed to sustainable development, especially as their raw material comes from sustainably managed forests. As the European Commission stated several years ago, “wood and timber products play an important role in mitigating climate change by absorbing and retaining carbon from the atmosphere” [49]. For a better comprehension of eco-friendly wood materials, and the recycling process, it becomes required to understand the physical and chemical properties of wood and recycled materials, as well as the interactions between wood, recycled material, and adhesive and technological conditions [50].

### 4.1. Environmental Profile (EP) for a General Carpentry Material

Carpentry or carpentry-work-construction work on the manufacture of wooden structures and parts is characterized by less careful wood processing. Carpentry work includes work on the construction of wooden walls, façades. By carpentry, wood is meant here as building wood exterior cladding, such as panel units for a kinetic façade) as well as veneer and chip products. The energy consumption for manufacturing is modest. As wood absorbs CO_2_ during growth and is therefore considered a CO_2_-neutral material, there are obvious environmental benefits from using it [51]. It has clear growth rings due to the large color difference between light springwood and dark autumn wood, selected as the color for the kinetic façade. With constructive wood protection, the use of heartwood and regular surface treatments, doors, and pine windows can last 90–120 years, and in a dry environment, the wood lasts 120–1000 years [52]. Depending on the environmental profile (EP) of 1 m^2^ of this material, with a thickness of 21 mm, where the estimated cycle life is 50 years, wood has a modest thermal conductivity [53].

### 4.2. Larix Wood Cladding

Larix belongs to a fast-growing and durable species: some of them live 700–900 years. A coniferous tree, up to 50–80 m high, shedding foliage for the winter, light-loving, and frost-resistant, grows throughout the Northern Hemisphere of our planet. Deciduous forests can withstand temperatures down to −60 °C [54]. Larix is among the hardest and heaviest coniferous species, with a very large heartwood proportion. The crucial environmental force of larch wood is that the heartwood does not need impregnation for exterior use. Larix wood is durable and very difficult to ignite. Its biggest physical and aesthetic weakness is the appearance of large and rather dark lumps. The heartwood has poor permeability and therefore moisturizes only to a limited extent by brief water exposure. Small dimensions, such as the environmental profile of 1 m^2^ Larix wood, with a thickness of 21 mm, where the estimated cycle life is 65 years, are recommended due to the lark’s tendency to twist and bend [53].

### 4.3. Cedarwood Cladding

Cedarwood is valuable because it does not rot in water, is not subject to fouling by algae and mollusks, and is not damaged by termites. Therefore, the red cedar is one of the most favorite options for the kinetic wooden façade (KWF). The wood of European origin is called thuja. European thuja is fast-growing and weaker than North American wood [55]. Western Red cedar grows mixed with other coniferous species in the Western US and Canada [56]. Cedar is a very light wood with modest compressive and bending strength, and therefore not suitable as a construction wood. However, it is among the most durable woods for outdoor use, partly due to a high content of essential oil with a moisture-repellent effect and partly due to the fungicidal Thujaplicin. In Northern Europe, cedar has been used more frequently during the 1990s, especially for exterior cladding of façades. Therefore, it is a suitable material for a wooden façade. The environmental profile (EP) of 1 m^2^ of cedarwood, with a thickness of 21 mm, has an estimated cycle life of 75 years [53].

### 4.4. Fiberboards

Paraffin is usually added to the wood pulp to improve the water-repellent properties of fiberboard boards. The boards’ strength can be increased by binding agents such as starch, rosin, and synthetic resins. Fiberboard boards are faced with natural wood veneer, paper, fabric, plastic, fiberglass, metal, and cork. The wood fiberboards are cut and applied to stains on one side to distinguish them from ordinary softwood boards [57]. The plates are wind- and moisture-tight but open to vapor diffusion. The wax-impregnated boards are cleaner to work with than similar asphalt-impregnated ones. The panels can emit very small amounts of formaldehyde over time, on a par with ordinary planed wood. Fiberboard with the above properties is used as a wind barrier behind a ventilated exterior cladding. Today the eco-friendly materials can be used frequently in a new generation of building materials, where the environmental aspects of the various board materials are just as different as their properties and applications. Eco-friendly fiberboard panels with acceptable physical and mechanical properties are in accordance with European standards [58].

Fiberboard is disposed of by incineration. Standard fiberboard boards are divided into two main classes:

- porous
- solid

In terms of its basic properties, the fiberboard material is comparable to wood since it retains all the useful qualities of wood, for example, strength, toughness; moreover, fiberboard is a warm material. Furthermore, the environmental profile (EP) of 1 m^2^ of fiberboard, with a thickness of 19 mm, has an estimated cycle life of 100 years [53].

### 4.5. Chipboard

Chipboard is a composite sheet material made by hot pressing of wood particles, mainly shavings, mixed with a binder of non-mineral origin with the introduction, if necessary, of special additives [59]. Particle board consists of pine or spruce shavings, possibly birch shavings such as cover layers, ureal glue, or phenol glue, as well as small amounts of wax. Slabs with a bulk density of at least 600 kg/m^3^ are used for building purposes [60]. Particle board is very sensitive to moisture, partly because wood chips absorb moisture to a much greater degree than, for example, defibrated wood material, and partly because the adhesives used are not or only partially moisture resistant. Thus, among the materials used for construction and furniture production, the chipboard takes an important place. The estimated cycle life is 50 years for the environmental profile (EP) of 1 m^2^ of chipboard, with a thickness of 21 mm [53].

### 4.6. Plywood

Plywood consists of glued together with thin wooden boards. The middle veneer layers are often spruce, possibly pine, and of Scandinavian origin. Good sorts are used, and the trunk’s best parts are used for veneers [61]. The outer cover layers can be beech, birch, or other wood types, depending on where and whether the plywood is used visibly. The purpose of cross-laying veneer layers is to produce a board that does not sag or shrink significantly and has great strength concerning its weight. Depending on the type of glue, plywood can be water- and boiling-resistant. Plywood treated with flame retardants and impregnated against fungus can also be produced. Plywood, also known as a wood-laminated sheet, is a multi-layer construction material created by gluing specially designed veneer [62]. Plywood is an inexpensive finishing material suitable for exterior wall decoration. It can be used for roughing under tiles or for finishing. Depending on the sheets’ brand, such a surface may require protection with varnish or paint, or not require additional processing at all. Plywood is used in construction because of its strength and durability, and versatility. Plywood can be made also from hardwoods, softwoods, bamboo, or a combination of different woods. The sustainability of plywood is determined not only by how the wood is being sourced but also by the manufacturing process [63]. The environmental profile (EP) of 1 m^2^ of plywood, with a thickness of 15 mm, has an estimated cycle life of 50 years [54].

## 5. Results

### 5.1. Material Passport (MP) for Wood Façade

The Material Passport application for the wood façade is investigated using the Mnaro et al. [35] model. General data, security, sustainability, usage and service, disassembly guide, reuse, history, and other details are required for passports. Product definition, manufacturer, device structure, usage recommendations and restrictions, technological assessment, structural efficiency, impact tolerance, durability against the xylophage species, water tightness, thermal and acoustic efficiency, and system reliability are the general data collected. The data contained in terms of protection measures include fire resistance and fire response assessment. The details gathered for sustainability include the implementation/execution protocol, transportation, assembly mechanism, and component assessment methods. The criteria for evaluating the wood frame system’s material and component characteristics are indicated in the National System of Technical Assessment documents, SİNAT-005/2017 [64].

### 5.2. The Indicators of the Environmental Profile (EP)

Sustainable development means increased welfare that considers the earth’s ecosystems and the inventory of renewable and non-renewable natural resources. Therefore, environmental evaluations of the built environment are becoming increasingly common [65]. An environmental profile is shown for the buildings’ materials and constructions per m^2^ floor area for buildings. All results shown in the environmental profile are expressed as annual values. In the calculation process, each material’s environmental impact is divided by its estimated lifetime and then summed up as part of the home’s total environmental impact. An environmental profile is also displayed, which compares the annual environmental impact per m^2^ of floor area from the building’s heating and building materials. This profile includes only energy consumption and the greenhouse effect. Here you can see how large a share of the total environmental impact is due to the materials. Finally, a layer cake diagram is shown, which compares the greenhouse effect’s environmental impact on the building’s wooden façade. BEAT 2000 is a suitable tool that can be used immediately for energy and environmental assessment of any environmental building analysis. It has expanded the database, especially for alternative energy-saving solutions, and the materials included could reduce the time consumption by defining the untraditional and unusual building parts that appear during renovation.

**Figure 2:**
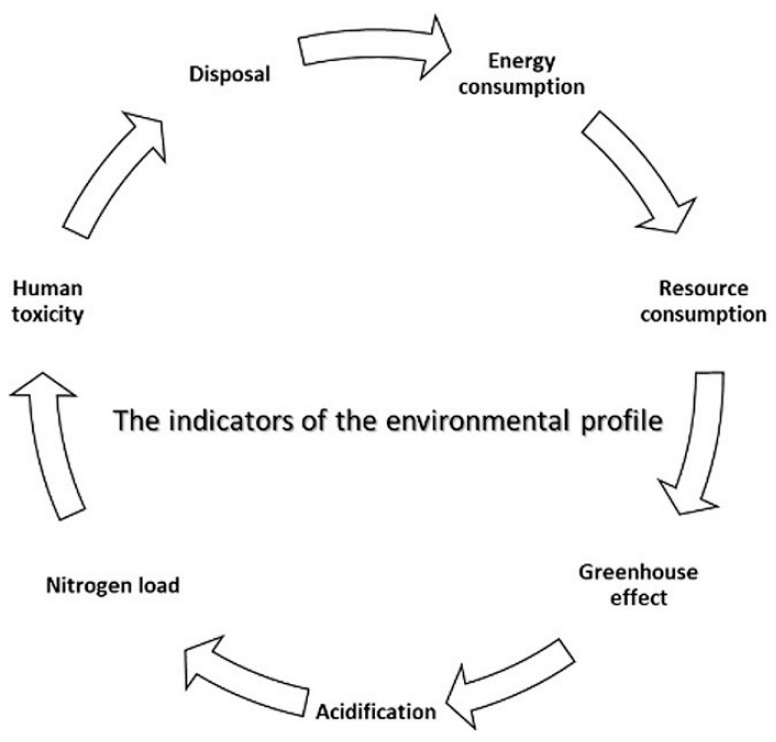
The environmental profile indicators.

The environmental profile (EP) consists of seven environmental indicators (see Figure 2) covering all significant physical environmental impacts and effects. Newer constructions are assumed to have environmental advantages and are considered to have a future in the European market. It was chosen to focus on climate screen constructions, primarily kinetic wooden façades, representing the most important component of a typical building and the most environmentally damaging part of a building. The building façade is also a central part of the architectural expression. All wooden façades have U-values related to Building European Regulations 2020 and Building Regulations for small houses 1998. All building façades have a U-value of 0.20, while the wooden window and glass façade have a U-value of 1.65. All roofs have a U-value of 0.15. For all constructions, an environmental profile (EP) for 1 m^2^ of the building in the analysis is shown. All results shown in the environmental profile are expressed as annual values, i.e., in the calculation of the environmental profile (EP), the environmental impact of each type of wooden material is divided by its projected lifespan in the existing structure and then added together as part of the overall environmental effect of the concept. Besides, a layer cake diagram is shown, which compares the environmental impact in the form of a greenhouse effect, distributed on the parts of the construction in analyzing:

### 5.3. Resource Consumption

**Figure 3:**
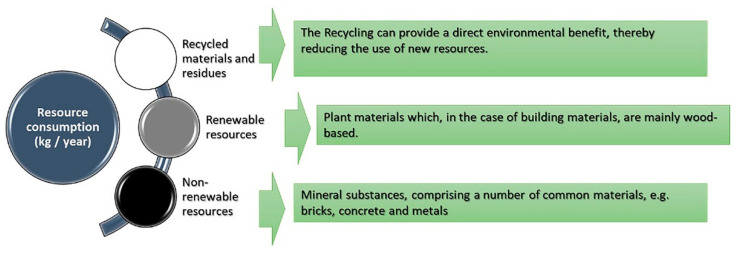
The environmental profile (EP) shows the environmental impact of Resource consumption.

The practice of sustainable building refers to various methods in the process of implementing building projects that involve less harm to the environment [35]. It is important to make use of natural resources. They are used as input in production and consumption and form increased safety [66]. The increasing environmental damage require urgent action to reduce environmental degradation [67]. Resource consumption represents an essential factor in the environmental profile (EP). It is about the consumption of non-renewable, or, less often, renewable, resources [68]. It explains the environmental impact of resource consumption (see Figure 3).

**Figure 4:**
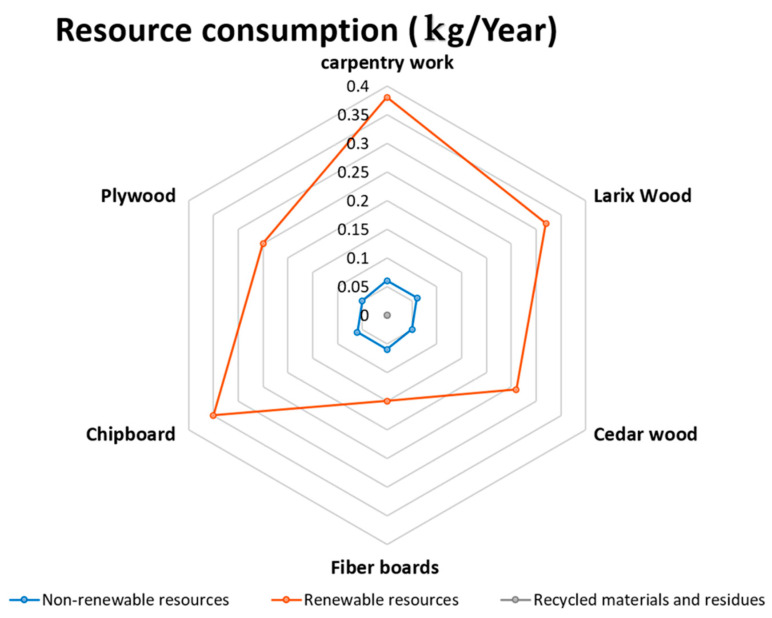
The impact of resource consumption on the selected façade wooden material environmental [68,69,70,71,72].

The environmental consequences of resource use may include a lack of energy, increased area consumed, and risks associated with the extraction or cultivation process. The results of applying this indicator to the five chosen wooden façade materials are seen in the diagram below (see Figure 4).

### 5.4. Energy Consumption

**Figure 5:**
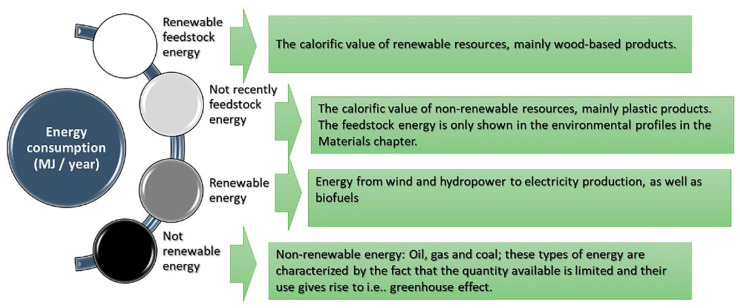
The environmental indicators show the environmental impact of resource consumption.

The environmental indicator shows the environmental impact of resource consumption (see Figure 5).

**Figure 6:**
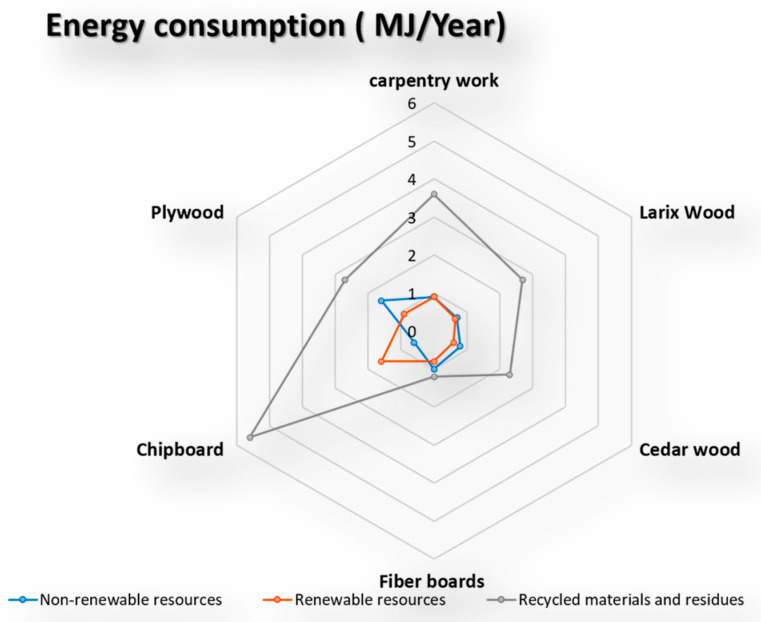
The impact of energy consumption on the selected façade wooden material [68,69,70,71,72].

The environmental consequences of resource use may include a lack of energy, an increased area consumed, and risks associated with the extraction or cultivation process. The results of applying this indicator to the five chosen wooden façade materials are seen in the diagram below (see Figure 6).

### 5.5. Greenhouse Effect (×1000/CO_2_/Year)

**Figure 7:**
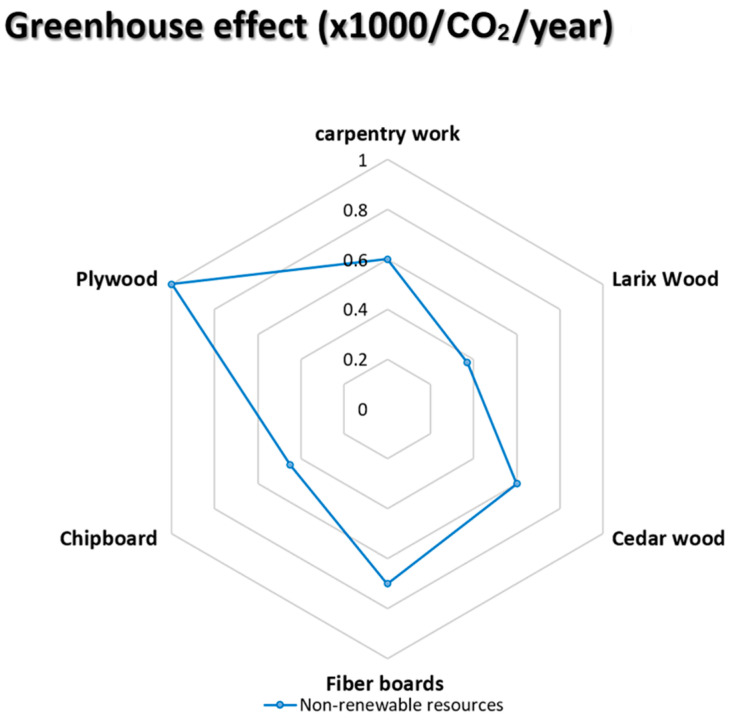
The impact of the greenhouse effect on the selective façade of wooden material [68,69,70,71,72].

The environmental impact of greenhouse gas pollution is the third predictor of the environmental profile. The human-caused greenhouse effect is caused mainly by the release of fluorinated greenhouse gases (F-GHGs) and other greenhouse gases, including nitrous oxides (NO_X_), methane (CH_4_), and carbon dioxide (CO_2_), that trap heat that would otherwise reflect from the planet to space. They are currently contributing to the warming of the atmosphere. The results of applying this indicator to the five chosen wooden façade materials are seen in the diagram below (see Figure 7).

### 5.6. Acidification (gSO_3_/Year)

**Figure 8:**
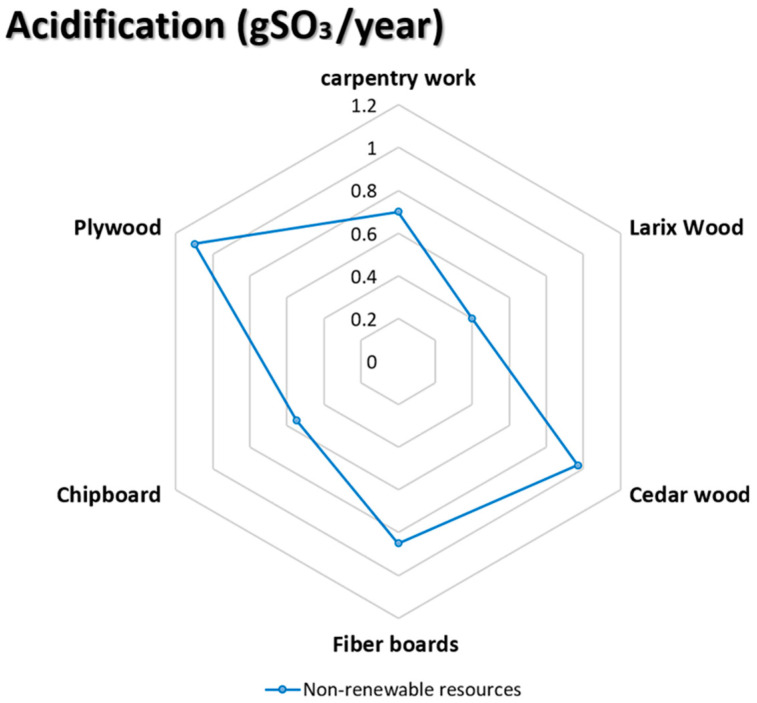
The impact of acidification on the selective façade wooden material [68,69,70,71,72].

The fourth indicator in the environmental profile (EP) depicts the environmental impact of acidifying compounds (particularly sulfur dioxide and nitrogen oxides), attacking plant leaves and needles and acidifying the soil. The results of applying this indicator to the five chosen wooden façade materials are seen in the diagram below (see Figure 8).

### 5.7. Nitrogen Load (gNO_3_/Year)

**Figure 9:**
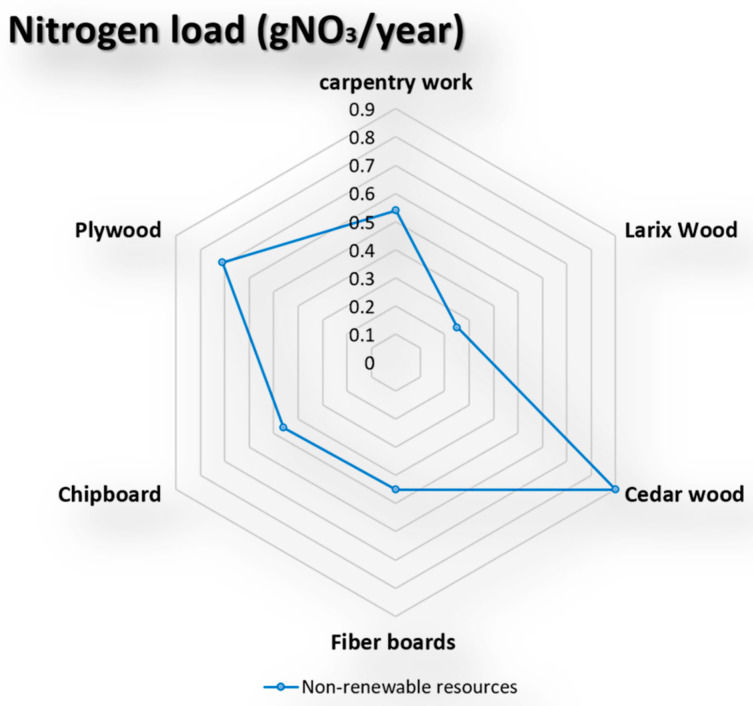
The impact of nitrogen on the selective façade wooden material [67,68,69].

The fifth measure in the environmental profile (EP) represents the impact of nitrogen- or phosphorus-containing compounds on the environment. They will lead to the expansion of algae or plants to get out of control, which is harmful to the environment. Applying this indicator to the selected five wooden façade materials is shown in the below diagram’s results (see Figure 9).

### 5.8. Human Toxicity (m^3^/Year)

**Figure 10:**
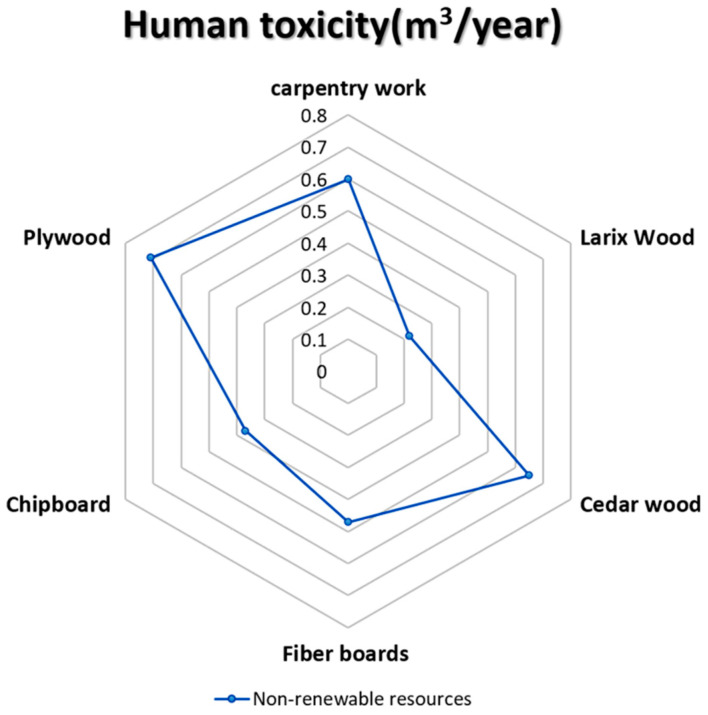
The impact of human toxicity on the selective façade wooden material [68,69,70,71,72].

The sixth predictor of the environmental profile (EP) indicates that pollutants with acute and permanent harmful effects on humans have an ecological impact. Contaminants are released into the receiving environment at the life cycle of goods, facilities, and systems, such as air, water, and soil. The human-health toxicity feature is described as DALY per kg of chemicals released into a given environment [72]. Emission inventories of various materials will include hundreds of chemicals, which could cause adverse effects to people and habitats. Applying this indicator to the selected five wooden façade materials is shown in the below diagram’s results (see Figure 10).

### 5.9. Disposal (kg/Year)

**Figure 11:**
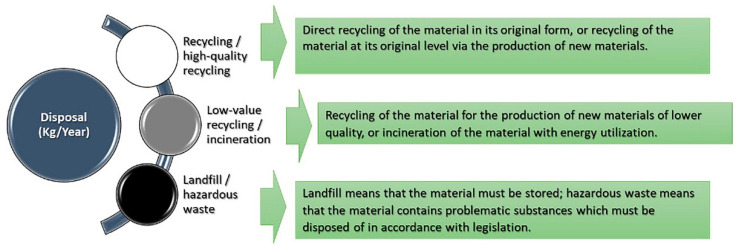
The environmental indicators show the environmental impact of the disposal.

The last indicator of the environmental profile (EP) shows the building, construction, or material when its service life is over (see Figure 11).

**Figure 12:**
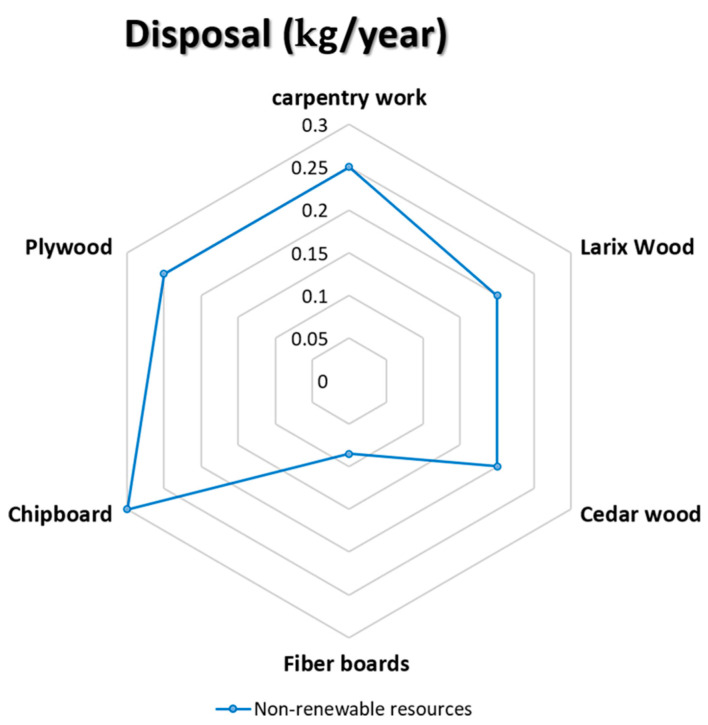
Shows the impact of disposal on the selective façade wooden material [68,69,70,71,72].

**Figure 13:**
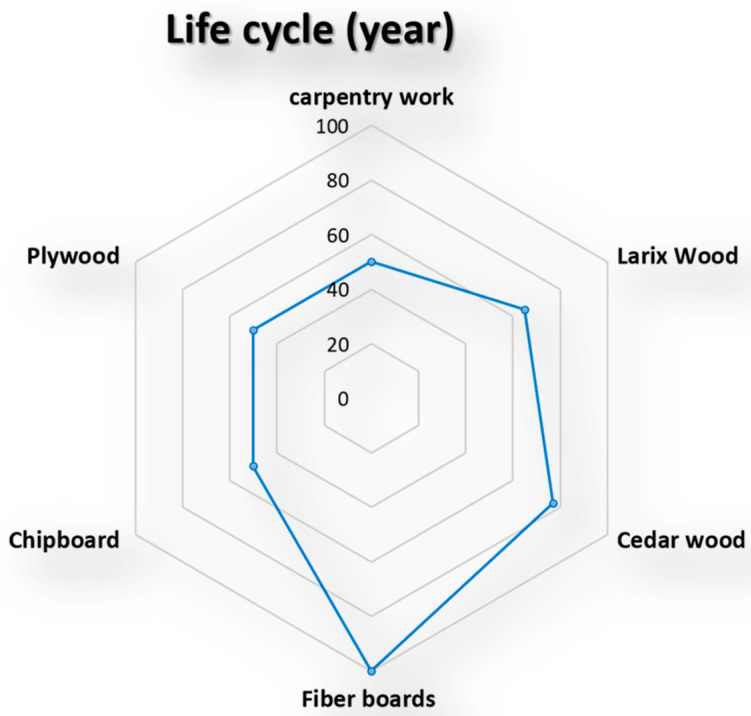
The life cycle façade wooden material [68,69,70].

Applying this indicator to the selected five wooden façade materials is shown in the below diagram’s results (see Figure 12 and Figure 13).

## 6. Discussion

Reserves and renovation speed of materials create limitations in accelerating the useability process. So, there has to be a new creation of it for a specific material to substitute the used one. For example, there are big reserves of natural stone but very low renovation speed; on the contrary, there are limited reserves of wooden materials but very high renovation speed.

The life cycle of a product, applied to the construction area, is all the ways it follows from the acquisition of the raw materials (resources) from nature to make it, passing through its processing, packaging, transportation, installation, use, maintenance, deconstruction, or demolition, obtaining a waste that we can directly reuse, recycle, or dispose of in a controlled landfill [73]. Then, of course, the cycle is closed when we do not waste anymore, but the raw material we had at the beginning, or another material, was equally useful.

Due to the environmental problems experienced today, the trend towards sustainable or recyclable materials in the construction sector has increased, and studies on ecological structure concepts have increased. Wood material is also preferred because it is natural, easy to handle, durable, and easily used with other materials. However, today’s comfort conditions have changed; new construction technologies have been developed; the number of floors of buildings has increased; and wooden building materials have been insufficient for these conditions.

The construction sector is lagging when it comes to digitization in comparison to other industry sectors. However, the rapid innovation and change in information technology offer an immense opportunity to implement a circular economy. With the increasing complexity and the substantial majority of materials and products in a building, digitization, process automation, and data standards need to be a prerequisite rather than an exception, besides the revolutionary advantages of digital technology for building and operational processes and materials passports [74].

Digital processes must gather, process, store, and use the massive quantities of data involved. Information stored in materials passports is only useful when the relevant actors can use it at the required time. Materials passports (MPs) need to be integrated into BIM to provide input data for reversible and circular design assessments [75]. BIM, which can be seen as a digital twin, will become a standard tool in the construction industry because it can store referencing and link data of individual components within a building over its life cycle. Materials passports and BIM should be seen in combination as they complement each other.

For effective use of IoT, the development of Artificial Intelligence (AI) plays a vital role. AI can assess information based on patterns (e.g., for information transfer) or when collecting data [76]. For example, an automated building façade scan can be interpreted (e.g., dimensions of windows) and analyzed. Furthermore, within the machine learning process, there is a possibility to identify material composition in an automated way in the future.

The architecture and engineering projects of the Circular Building highlight the transition from the industry-led linear economy paradigm of “take, make, waste” to the circular economy [77]. The project is the third in a series of projects they have worked on with Lewis Blackwell, Chief Strategy Officer of The Building Center in London. Smith constructed the Wikihouse in 2014 and the A House for London in 2015. Both projects were designed to explore the different technologies and construction methods that emerged at that time. The theme of Wikihouse was the open-source design and digital manufacturing, while A House for London was the modular building and housing crisis, and the issues of materials, resources, and waste in the construction industry were addressed [78]. The construction industry provides the necessary infrastructure, offices, and homes for our cities and neighborhoods and undeniably impacts the environment. According to calculations, 10 million tons of the approximately 20 million tons of waste generated in London in 2008 are from construction [79]. As the construction industry practices develop, what to do with the increasing waste is a big problem, as well as simultaneously extracting the mines used in the sector and transforming them into building materials, the environmental damages caused by the energy consumed. The Circular Building idea arises from this point. Cyclic Building is not a circular structure; the adjective “cyclic” describes the journey of materials. Ellen MacArthur, who had been offshore for 71 days, broke the record for traveling worldwide by her sailing boat on February 7, 2005, when she arrived at Ushant in Breton, France [80]. She embarked on another circular journey in September 2010 when she founded the Ellen MacArthur Foundation, promoting the “circular economy.” A circular economy reproduces and reuses materials just as in nature, unlike the “take, make, waste” model of our linear economy, which is based on consuming resources. It transforms waste into food thanks to pre-planned design processes. The fundamental philosophy has to maintain goods at their maximum value for as long as possible so that structures and facilities can be used for as long as possible. The “biosphere” and “technosphere”, named after Swiss architect Walter Stahel [81] and based on the “cradle to cradle” concept, illuminated by Michael Braungart as a chemist, and William McDonough as an architect, bring the model of material circulation to existence [82].

The idea of a prototype building that is planned to bring together many components from the construction industry, then disassemble the parts and return them to the supply chain, created a new vision for materials engineering, and recycling has been made possible by digital technologies [83]. Suppliers would be responsible for regenerating all of the materials and making them available. The materials with the lowest possible energy use and low carbon impact were chosen. A complete digital model was created and integrated the material passport idea into the building construction. During the dismantling of the building, footprints of every material used were recorded. Thus, the building became a material resource, an archive to be used in the future. Buildings are described as material banks in the context of the material passport. Instead of being dismantled during use, buildings’ pieces are properly disassembled and reused or recycled at the same or even higher quality standards than the conventional make-a-waste scheme. Material passport promotes a waste-free economy. Materials passports facilitate circular business models by identifying materials and displaying their circular pathways.

Suppliers, designers, engineers, and end-users are now able to access information from a digital portal. The aim is to create materials with continuous cycles of use and reuse. For example, in the maintenance cycle, products are retained to optimize their usage time, and the value for recovery is assured in the reprocessing and energy production loop. The refurbishment loop allows biosphere materials to be safely cascaded into new products until they ultimately re-enter the biosphere through incineration or composting. Many items are not yet planned to be used circularly. The passports portal offers a feedback loop to refine their content, product design, and facilities for improved circular use materials passports, facilitating adapting to a circular economy. Engineering and design processes in the circular economy require close collaboration, especially with material suppliers. People find this idea very attractive; they are interested in the process itself and the ideas it came up with, and they started using the modular structure we currently use, digital fabrication, the use of materials from cradles to cradles.

Stewart Brand’s book entitled “How Buildings Learn” is a fundamental source on this topic [84]. American author and visionary Stewart Brand is also the editor of the famous 1968 work “Whole Earth Catalog” [85], which illuminated the idea of Shearing Layers, developed by architect Frank Duffy [86], in his 1994 book entitled “How Buildings Learn: What Happens After They Are Built” [87]. In this approach, a building is not considered a single entity, but a structure composed of elements that transform according to different timelines. The layers of a building are called Six “s”: Site (location); Structure; Skin (surface); Service (electricity, plumbing, heating); Space Plan (layout); and Stuff (items), in other words, other stuff such as furniture that belongs to homes. Different spatial elements have different timelines [84].

The items of the interior can also be designed differently. For example, rug suppliers such as Desso offer the opportunity to rent a rug; the rugs would be returned at the end of the time [88]. They also produce a new rug using the same material. It is possible to adopt the same model for electrical products. Regarding Phillips’s “pay as you burn” model for lighting, the user (not the consumer) pays for the light he uses instead of the material. There is no limit to re-evaluating lighting elements. The project also benefits from traditional materials that have undergone chemical applications. A wood material called Accoya is the façade cladding. Accoya has become a game-changer material. Accoya, produced from a fast-growing softwood, is treated with acetylation [86]. It is akin to drying wood pickles. Acetylation similarly hardens the wood and stops the moisture movements in it. It becomes durable and can be used repeatedly. It is a great material that is as hard as wood.

Circular economy practices are spreading, although not yet mainstream. If the construction industry did not support, it would not have made that much impact. It is an important step forward in construction and engineering and a very exciting process; innovators will probably continue to look for ways to disseminate for the next decades. This approach will completely change where the materials are gathered, what they are used for, and how they are utilized. The novelty of this study is to conduct a comprehensive, environmentally comparative analysis of the vast of building materials and define the smart kinetic façade role in modern building design depending on environmental profile (EP) inquiry, regarding the idea of integrating environmental sensitivity into the material passport phenomenon. The results show that considering materials’ environmental sensitivity in terms of resource consumption, energy consumption, greenhouse effect, acidification, nitrogen load, human toxicity, disposal, and life cycle, respectively, as a model within the material passport (MP) concepts, will significantly contribute to the use of the right building material at the right cost.

## 7. Conclusions

Wood is certainly the oldest natural material, renewable, easily recyclable, and it can store carbon dioxide, making high wood buildings a solution capable of meeting key sustainability goals. Moreover, since the early 20th century, thanks to the attention and dissemination of concepts related to the environmental sustainability of processes and production, they are studied and appreciated regarding other values about the ecosystem balance and the perceived environmental comfort in buildings made of wood. Furthermore, while wood is used depending on the size of the tree used in traditional buildings, it can be prepared in industrially desired sizes in today’s conditions. This situation has increased the usage area of wood and has made it a material above the standards of other materials we use in today’s needs.

Increasing ecological awareness, growing expectations for the health and comfort of home environments, and developing interesting new products from the wood industry are the basis for modern construction designs in the urban context. We take a holistic view of energy consumption and the material cycle in the construction industry, and we see that wood offers many advantages. For example, wood binds carbon dioxide during the growth phase and protects it for years even when it becomes a wood-building material, thus preventing carbon dioxide from entering the atmosphere again.

The study explores the possibilities to generate worth in the human-made facilities by integrating the building material passport and the wooden kinetic façade (WKF), to incorporate circular qualities into field value chains. The importance of data analysis to invent and add value is shown in this tool. Thus, to optimize materials by regeneration and recycling, the notion of waste is revised. The reintegration of materials into the innovative business models is crucial to support LCA, also end-of-life studies. Many barriers in the civil construction field to applying the BMP instrument include systemic consideration of the value chain and flows, resulting in improved cooperation among stakeholders and public assistance, emphasizing regulations and fiscal incentives necessary for a circular economic transition.

Instead of a linear system, a transformation into a circular economy will develop, and new features will appear. Establishing take-back systems is required to provide incentives for participation with the information exchange and innovative business models. To obtain the maximum benefit from materials passports for a circular economy, exchanging relevant, up-to-date information at the right time is key to a functioning value chain.

The availability of material data is a core aspect of a functioning circular economy. As buildings and components have long lifetimes and can have multiple changes of ownership and responsibilities, the data need to be kept up to date and passed on to the relevant actors systematically. A circular supply chain is only as strong as its weakest link, which requires incentives to ensure the participation of all parties. We need to start acting now in implementing the necessary steps in the building industry and its supply chain because establishing a circular economy is a prerequisite for sustainable development towards a sustainable and more circular future.

## Data Availability

Not applicable.
